# Notch Signaling Pathway Is Activated in Motoneurons of Spinal Muscular Atrophy

**DOI:** 10.3390/ijms140611424

**Published:** 2013-05-29

**Authors:** Víctor Caraballo-Miralles, Andrea Cardona-Rossinyol, Ana Garcera, Laura Torres-Benito, Rosa M. Soler, Lucía Tabares, Jerònia Lladó, Gabriel Olmos

**Affiliations:** 1 Cellular Neurobiology Group, IUNICS and Department of Biology, University of the Balearic Islands, Palma de Mallorca 07122, Spain; E-Mails: victor.caraballo.uib@gmail.com (V.C.-M.); andrea.cardona@uib.es (A.C.-R.); jeronia.llado@uib.es (J.L.); 2 Neuronal Signaling Unit, Department of Basic Medical Sciences, University of Lleida-IRBLLEIDA, Lleida 25198, Spain; E-Mails: ana.garcera@udl.cat (A.G.); rosa.soler@cmb.udl.cat (R.M.S.); 3 Department of Medical Physiology and Biophysics, School of Medicine, University of Seville, Seville 41009, Spain; E-Mails: latorres@us.es (L.T.-B.); ltabares@us.es (L.T.)

**Keywords:** SMA, Notch, NICD, Jagged1, Delta1, Neurogenin 3, astrocyte, motoneuron

## Abstract

Spinal muscular atrophy (SMA) is a neurodegenerative disease produced by low levels of Survival Motor Neuron (SMN) protein that affects alpha motoneurons in the spinal cord. Notch signaling is a cell-cell communication system well known as a master regulator of neural development, but also with important roles in the adult central nervous system. Aberrant Notch function is associated with several developmental neurological disorders; however, the potential implication of the Notch pathway in SMA pathogenesis has not been studied yet. We report here that SMN deficiency, induced in the astroglioma cell line U87MG after lentiviral transduction with a shSMN construct, was associated with an increase in the expression of the main components of Notch signaling pathway, namely its ligands, Jagged1 and Delta1, the Notch receptor and its active intracellular form (NICD). In the SMNΔ7 mouse model of SMA we also found increased astrocyte processes positive for Jagged1 and Delta1 in intimate contact with lumbar spinal cord motoneurons. In these motoneurons an increased Notch signaling was found, as denoted by increased NICD levels and reduced expression of the proneural gene neurogenin 3, whose transcription is negatively regulated by Notch. Together, these findings may be relevant to understand some pathologic attributes of SMA motoneurons.

## 1. Introduction

Spinal muscular atrophy (SMA) is a neurodegenerative disease inherited in an autosomal recessive manner that affects alpha motoneurons in the spinal cord, and causes muscular atrophy of proximal limb and trunk muscles, paralysis, and in the most severe cases, death [1,2]. SMA is caused by the homozygous deletion or specific mutations of the Survival Motor Neuron 1 (SMN1) gene, which results in reduced dosage of full-length SMN protein [3]. The deletion of SMN1 homologs in other animals is lethal at early embryonic ages [4]; however, the human genome contains a variable number of copies of the SMN2 gene that produces about 90% of a highly unstable, truncated protein, called SMNΔ7 due to defective mRNA maturation, and only 10% of normal (full-length) protein [5]. Thus, the severity of the disease depends on the number of copies of SMN2 gene [6].

Notch signaling is a cell-cell communication system well known as a master regulator of neural development [7–9]. Four Notch receptors (Notch1–4) and five ligands (Jagged1 and 2; Delta-like1, 3 and 4) have been identified in mammals [10]. Upon ligand binding, a series of cleavage events culminate in the proteolytic cleavage of the transmembrane Notch receptor by the γ-secretase, giving rise to the Notch intracellular domain (NICD), which is translocated into the cell nucleus. Canonical Notch signaling involves the binding of NICD to DNA-binding cofactors and the subsequent activation of the transcription of target genes [11,12]. The most studied Notch targets are the Hairy and Enhancer of Split (Hes) genes. Hes1 negatively controls the expression of a series of proneural genes, including Neurogenin 3 (Ngn3), involved in neuritogenesis [13–15]. During the development of the central nervous system in vertebrates, the nascent neurons, by expressing Delta1, deliver lateral inhibition to the Notch1-expressing progenitors in contact with them, so as to prevent these progenitors from differentiating prematurely into neurons and from expressing Delta1 [16]. In addition, Notch expression persists throughout the adult brain in differentiated cells [17,18]. Aberrant Notch function is associated with several developmental neurological disorders and neurodegenerative diseases (reviewed by [19]), and increased expression of Notch has been described in Alzheimer’s disease, Pick’s disease and Down syndrome [20,21]; however, the potential implication of Notch pathway in SMA pathogenesis has not been studied yet.

Several findings suggest a potential implication of the Notch system in SMA pathology: Notch1 (hereafter referred to as Notch) as well as SMN share common functions in the regulation of neurite outgrowth [13,22–25], cell migration [26,27], axon guidance [28,29] and neuromuscular junction maturation [30,31]. In addition, Notch signaling functions in astrocytes as an inducer of characteristic elements for reactive gliosis [32,33], and reactive gliosis has also been described in different types of human SMA [34–36] and in the ventral spinal cord of SMA mice [37]. In this sense, we hypothesized that SMN depletion could be related to an increased activation of the Notch signaling pathway in astrocytes. Thus, we first studied *in vitro*, in the U87MG astroglioma cell line experimentally depleted of SMN, the immunoexpression of Notch, its active intracellular domain (NICD) and its ligands (Jagged1 and Delta1). Then, in an *in vivo* model of SMA, the SMNΔ7 mouse model, we also studied the expression of Notch ligands in reactive astrocytes in relation with the potential activation of the Notch signaling in the neighboring spinal cord motoneurons.

## 2. Results

### 2.1. Increased Notch Signaling in U87MG Astroglioma Cells Depleted of SMN

**Figure 1:**
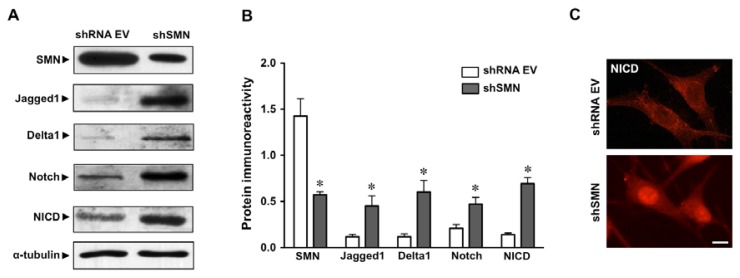
Activation of Notch pathway in U87MG astroglioma cells by Survival Motor Neuron (SMN) depletion. (**A**) Western blots showing SMN, Jagged1, Delta1, Notch and anti-cleaved Notch1 (NICD) immunoreactivities in U87MG cells after transduction with lentiviruses containing the empty vector (shRNA EV) or the shSMN constructs, as indicated. (**B**) Column bars indicate the immunoreactivity of each protein as determined by densitometric analysis (integrated optical density of each protein band *vs.* that of α-tubulin band), and are the mean ± SEM of three independent experiments. * At least *p* < 0.01 as compared to shRNA EV. (**C**) Immunocytochemistry showing increased NICD in the nuclei of cells transduced with shSMN construct as compared to that transduced with shRNA EV, as indicated. Scale bar = 10 μm.

Four days after lentiviral transduction of U87MG astroglioma cells with an shRNA sequence targeting SMN nearly a 60% reduction in SMN expression levels was found by western blotting, as compared to those transduced with shRNA EV (Figure 1A,B) and as previously described [27]. Then, the expression levels of four participants in the Notch signaling pathway, namely its ligands Jagged1 and Delta1, the Notch receptor and its active intracellular form (NICD) were studied by western blotting. The expression of these proteins was found significantly increased after SMN depletion. The Notch ligands Jagged1 and Delta1 increased their expression around five to six fold. The Notch receptor increased its expression around two fold, whereas the levels of its active form, NICD were found increased around four fold, as compared to shRNA EV (Figure 1A,B). Moreover, by performing immunocytochemistry in U87MG cells, increased NICD immunoreactivity was found in the nuclei of SMN deficient cells as compared to those transduced with shRNA EV (Figure 1C).

Together, these results indicated that SMN depletion in an astrocyte cell line was associated to an increased activation of the Notch signaling pathway. We therefore studied in an *in vivo* model, the SMNΔ7 mouse, if Notch ligands could also be increased on spinal cord astrocytes, as well as the potential effects on neighboring spinal cord motoneurons.

### 2.2. Increased Notch Signaling in Spinal Cord Motoneurons of the SMNΔ7 Mouse

**Figure 2:**
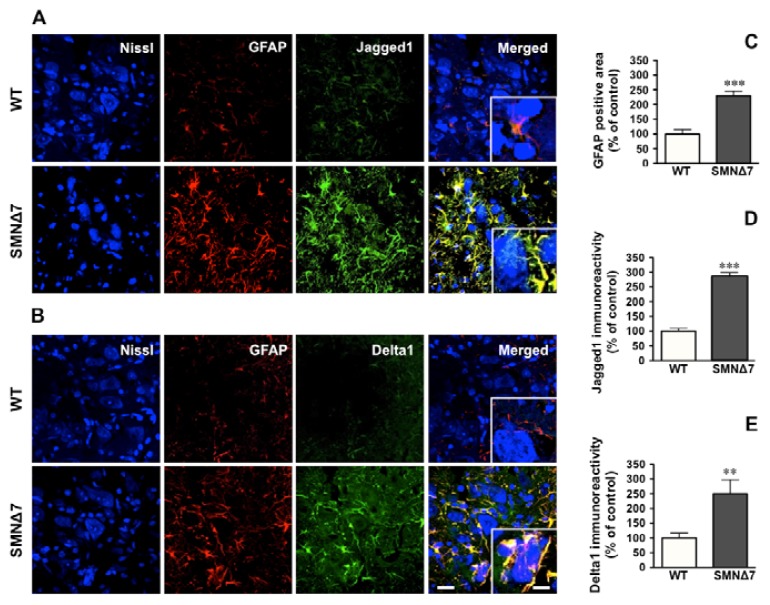
Astrocytosis and increased expression of Jagged1 and Delta1 in the ventral spinal cord of SMNΔ7 mice. (**A**,**B**) Representative images of anti-glial fibrillary acidic protein (GFAP) and Jagged1 (**A**) or GFAP and Delta1 (**B**) immunohistochemistry in the ventral horn of lumbar spinal cord of P11 wild-type (WT) and SMNΔ7 mice. Insets: astrocytes processes double labeled with GFAP and Jagged1 in (**A**) or with GFAP and Delta1 in (**B**) are in close apposition with motoneurons labeled with blue fluorescent Neuro Trace Nissl staining. Scale bars in (**B**) = 20 μm and 10 μm (inset), also apply to (**A**). (**C**) Columns represent the percentage of GFAP positive area in the ventral horn of lumbar spinal cord of P11 SMNΔ7 as compared to control (WT) mice and represent the mean ± SEM (error bars) of at least four mice for each group. *** *p* < 0.001 as compared to age-matched WT. (**D**,**E)** Columns represent the percentage of Jagged1 (**D**) or Delta1 (**E**) immunoreactivities in GFAP-positive cells (astrocytes) in the lumbar spinal cord of P11 SMNΔ7 as compared to control (WT) mice and represent the mean ± SEM (error bars) of at least four mice for each group. *** *p* < 0.001 and ** *p* < 0.01 as compared to age-matched WT.

In the SMNΔ7 mouse model of SMA, motor impairment is manifest at postnatal day 11 (P11) [38]. Immunostaining was performed for GFAP to visualize astroglia in the lumbar spinal cord of SMNΔ7 mice at this postnatal age. Quantification of the relative area of GFAP-positive structures within the ventral horn demonstrated a significant increase in this parameter in SMA mutants as compared to WT (Figure 2A–C). In SMA astrocytes the expression of the Notch ligands Jagged1 and Delta1 was found to be significantly increased (281 and 249 percent of increase; respectively) (Figure 2A,B,D,E). Spinal cord motoneurons were identified by their large bodies (>20 μm) when labeled with blue fluorescent Neuro Trace Nissl staining (Figure 2A,B). Astroglial processes with strong immunoreactivities for Jagged1 and Delta1 were found in intimate contact with motoneurons in SMA (Figure 2A,B, insets).

**Figure 3:**
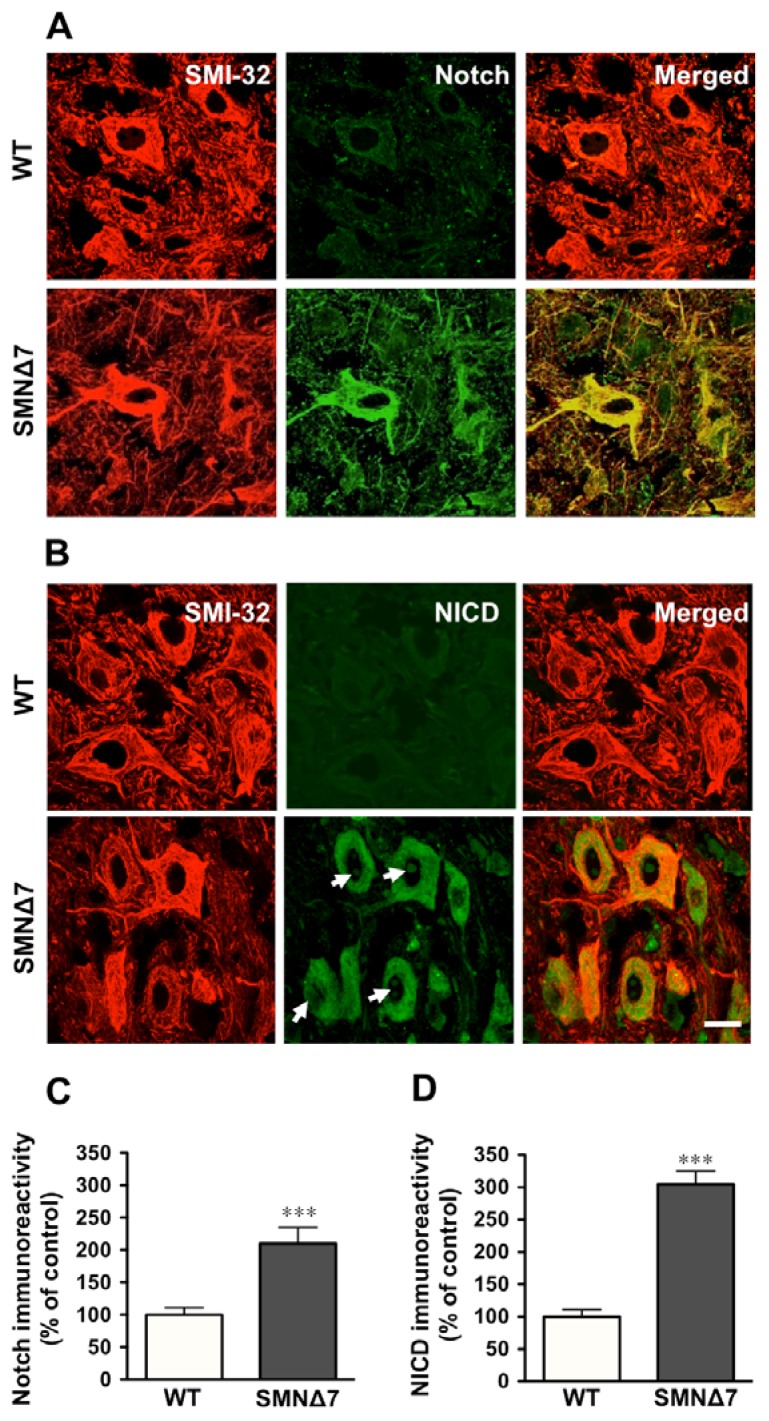
Notch signaling is activated in spinal muscular atrophy (SMA) spinal cord motoneurons. (**A**,**B**) Representative images of SMI-32 and Notch (**A**) or SMI-32 and NICD (**B**) immunohistochemistry in the ventral horn of lumbar spinal cord of P11 wild-type (WT) and SMNΔ7 mice showing that motoneurons are co-labeled with these antibodies (merge), and display increased Notch (**A**) or NICD (**B**) expression in SMNΔ7 mice. Arrows in (**B**) indicate the presence of NICD immunoreactivity in the nucleus of SMNΔ7 motoneurons. Scale bar = 20 μm applies to all photographs in (**A**) and (**B**). (**C**,**D**) Columns represent the percentage of Notch (**C**) or NICD (**D**) immunoreactivities in SMI-32 positive cells (motoneurons) in the lumbar spinal cord of P11 SMNΔ7 as compared to control (WT) mice and represent the mean ± SEM (error bars) of at least four mice for each group. *** *p* < 0.001 as compared to age-matched WT.

Activation of Notch receptor is induced via cell to cell contact-mediated binding of its ligands; thus, we examined if Notch pathway could be activated in spinal cord motoneurons of SMA mutants. A significant increase (215%) in the immunoreactivity of Notch receptor was observed co-localizing with that of SMI-32 antibody in lumbar spinal cord motoneurons of SMA mutants, as compared to age-matched WT animals (Figure 3A,C). Moreover, a significant increase (308%) in the immunoreactivity for NICD was found in SMA motoneurons (Figure 3B,D). Increased levels of NICD were detected in the perikaryon but also in the nucleus of these cells (Figure 3B, arrows).

**Figure 4:**
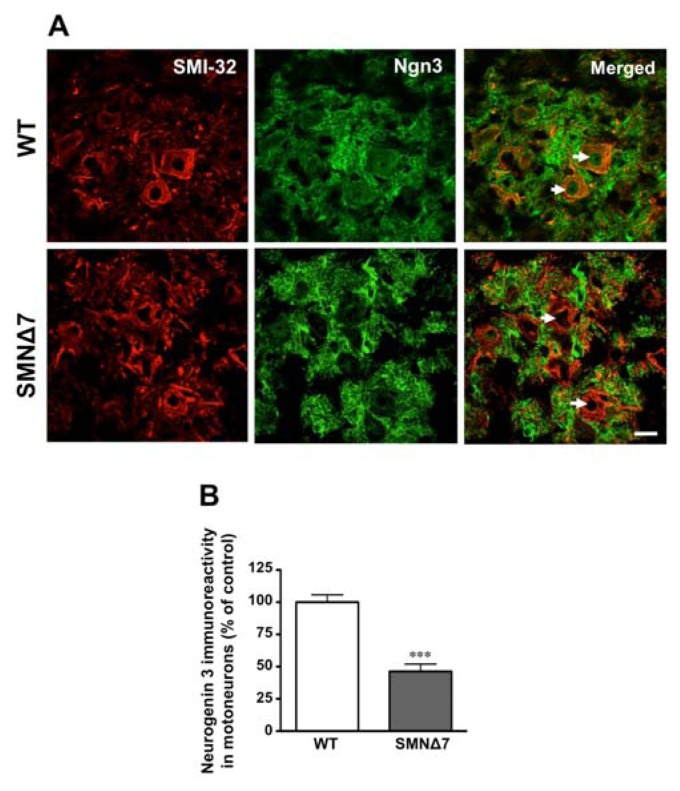
Decreased neurogenin 3 (Ngn3) expression in SMA spinal cord motoneurons. (**A**) Representative images of SMI-32 and Ngn3 immunohistochemistry in the ventral horn of lumbar spinal cord of P11 wild-type (WT) and SMNΔ7 mice showing that motoneurons are co-labeled with these antibodies (merge), and display decreased Ngn3 expression in SMNΔ7 mice. Arrows point to the nuclei of motoneurons to indicate the lack of Ngn3 immunoreactivity in SMNΔ7 mouse. Scale bar = 20 μm applies to all photographs in (**A**). (**B**) Columns represent the percentage of Ngn3 immunoreactivity in SMI-32 positive cells (motoneurons) in the lumbar spinal cord of P11 SMNΔ7 as compared to control (WT) mice and represent the mean ± SEM (error bars) of at least four mice for each group. *** *p* < 0.001 as compared to age-matched WT.

The activation of Notch signaling in postmitotic neurons results in the inhibition of the expression of Ngn3 [13]. Thus, to further test Notch signaling activation in SMA motoneurons, Ngn3 levels were studied in these cells. Ngn3 immunoreactivity was found to be located both in the nucleus and perikaryon of spinal cord motoneurons in WT mice (Figure 4A), by contrast, a significant reduction (54%) of Ngn3 immunoreactivity in motoneurons of SMA mutants was found, with absence of Ngn3 in their nuclei (Figure 4A,B). Thus, SMA motoneurons show increased NICD levels and reduced Ngn3 expression, confirming the activation of the Notch pathway in these cells.

## 3. Discussion

SMN deficiency induced *in vitro* in the astroglioma cell line U87MG resulted in an increase in the expression of the main components of Notch signaling pathway, as well as increased localization of NICD in cell nuclei. These results prompted us to explore in the SMNΔ7 mouse model of SMA the expression of Notch ligands in reactive astrocytes, and the potential activation of the Notch signaling in the neighboring spinal cord motoneurons.

Our results, indicating astrocytosis in the lumbar spinal cord of SMNΔ7 mice at P11, are in agreement with previous findings in which, in a more severe mouse model of SMA, ventral horn astrocytosis was detectable before and during spinal cord motoneuron death [37]. Interestingly, motoneuron loss has also been reported in the lumbar spinal cord of the SMNΔ7 mice at symptomatic stages [38]; thus, astroglial activation in SMNΔ7 mice may be associated with motoneuron pathology, as previously described in amyotrophic lateral sclerosis [39]. How reactive astrocytes affect motoneuron function in SMA has not been studied yet. Here, we report that reactive astrocytes in SMA display increased expression of the Notch ligands Jagged1 and Delta1. Although *in vitro* SMN depletion induced increased expression of Notch ligands in U87MG cells, suggesting a potential role of SMN as a repressor of Notch ligands expression in astrocytes, the mechanisms that regulate their expression *in vivo* in mature astrocytes are not fully understood. In this sense, the expression of Jagged1 is increased in astrocytes under an inflammatory environment [32] and it has been proposed the involvement of inflammatory pathways in SMA [40]. Moreover, the cytokine transforming growth factor β1 (TGFβ1) is directly implicated in the up-regulation of Jagged1 expression in astrocytes [41]; interestingly, disruption of TGFβ signaling is an important molecular event in the pathogenesis of several motoneuron diseases [42]. In addition, Jagged1 has an important role promoting astrocytosis [43] through the induction of GFAP gene expression [32]; thus, it can be proposed that the observed increase in GFAP immunoreactivity in the ventral horn of our SMA mutants may be also a result of the increased expression of Jagged1 in astrocytes.

It has been demonstrated that Jagged1 induces Notch signaling in adjacent cells through a cell-to-cell relay [44]. As astrocyte processes expressing high levels of Notch ligands were observed in intimate contact with spinal cord motoneurons, we addressed if the Notch pathway could be activated in these cells. Immunoreactivity for the active form of Notch (NICD) was found increased both in the nucleus and perikaryon of motoneurons from SMNΔ7 mice, demonstrating an activation of Notch signaling in SMA motoneurons. A similar pattern of NICD distribution has been described in hippocampal neurons in response to activity; in these cells a parallel increase in Notch receptor expression was also reported [45]. In agreement with these findings, we also found an increase in Notch receptor expression in SMA motoneurons. As NICD is a relatively unstable fragment of Notch [46], it has been proposed that positive feedback loops occur in Notch system in which Notch ligands maintain increased Notch receptor expression in cells undergoing Notch signaling [44,47]. Thus, our results suggest that SMN depletion *in vitro* in an astroglioma cell line, and *in vivo* in the SMNΔ7 mouse is associated to an increased expression of Notch ligands in astrocytes and that these cells may activate Notch signaling in adjacent motoneurons.

Ngn3 is a protein whose expression is negatively regulated by Notch signaling [13–15]. Our results demonstrating reduced Ngn3 expression in SMA motoneurons further indicate that Notch signaling is abnormally active in these cells. In WT motoneurons Ngn3 immunoreactivity was found both in the perikaryon and the cell nucleus. Previous studies have shown that Ngn3 immunoreactivity is also located in the perikaryon and neurites of hippocampal neurons, however, during the differentiation process its immunoreactivity progressively increases in the nucleus [48]. As Ngn3 functions as a transcriptional regulator, our results indicating a total absence of Ngn3 immunoreactivity in the nucleus of SMA motoneurons arise important repercussions in the context of SMA pathogenesis. Ngn3 has been demonstrated to promote neurite outgrowth [13]. In this sense, lack of axonal outgrowth has been described in some spinal cord motoneurons of human SMA [49] and, in murine models, it has been reported an important denervation of some muscles (up to 50% in intercostal muscles), together with a functional deficit and arrest of postnatal development of neuromuscular junctions, showing clusters of unoccupied acetylcholine receptors [31,50]. These findings indicate that impairment/lack of motor nerve terminals in SMA could be related to Ngn3 deficit in spinal cord motoneurons. In PC-12 cells, SMN deficiency also resulted in neuritogenesis impairment in NGF-differentiated cells; however, the effect was related to an up-regulation of the RhoA/ROCK pathway [23]. Also, we reported that in U87MG cells SMN deficiency resulted in impaired cell migration by altering actin cytoskeleton through the activation of RhoA/ROCK [27]. Interestingly, Notch signaling has also been found to activate this pathway to regulate actin dynamics [51] and thus, to inhibit neurite extension [52]. In addition to an impaired neuritogenesis, increased Notch activation in spinal cord motoneurons may predispose them to apoptosis, as demonstrated in cortical neurons in response to ischemic stroke [53].

Besides to its roles in neurons and astroglia, Notch has profound functions in other cell types in the brain, including microglia [54], oligodendrocytes [55] and endothelial cells [56]; thus, further studies are needed towards understanding the role of Notch system in SMA.

## 4. Experimental Section

### 4.1. U87MG Cell Culture and Transduction

U87MG human astroglioma cells were a gift from Dr. Priam Villalonga (IUNICS, University of the Balearic Islands, Palma de Mallorca, Spain). U87MG cells were subconfluently grown and passaged, routinely tested for mycoplasma contamination and subjected to frequent morphological tests and growth curve analysis as quality-control assessments. U87MG cells were grown in Dulbecco’s Modified Eagle’s Medium (DMEM) supplemented with 2 mM l-glutamine and 5% heat-inactivated fetal calf serum in a humidified incubator at 37 °C with 5% CO_2_.

To reduce SMN expression, RNA interference experiments were employed using lentiviral particles, as previously described [27]. Briefly, constructs were generated in pSUPER.retro.puro (OligoEngine; Seattle, WA, USA) using specific oligonucleotides targeting SMN sequence, indicated by capital letters and as previously described [57]; forward: gatccccCGACCTGTGAAGTAGCTAAttcaagagaTTAGCTACTTCACAGGTCGttttt and reverse: agctaaaaaCGACCTGTGAAGTAGCTAAtctcttgaaTTAGCTACTTCACAGGTCGggg. For lentiviral transduction, cells were plated at a density of 25,000 cells/mL in 6-well plates and 3 h later medium was replaced with medium containing lentiviruses (2 TU/cell) carrying shRNA empty vector (EV) or shSMN. The medium was replaced with fresh medium 24 h later and infection efficiency was monitored in each experiment by direct counting Green Fluorescent Protein (GFP)-positive cells. Cells were grown for 4 additional days before sample collection for western blotting or immunocytochemistry assays.

### 4.2. Western Blotting

U87MG cells were rinsed in ice-cold PBS and lysed with 50 mM Tris HCl, pH 6.8, 150 mM NaCl, 1 mM EDTA, 1% Triton X-100 containing a cocktail of protease inhibitors (Complete Mini; Roche Pharmaceutical, Basel, Switzerland). Lysates were sonicated and proteins quantified by means of the DC Protein Assay from Bio-Rad Laboratories (Hercules, CA, USA). Protein equivalents from each sample were resolved in SDS-polyacrilamide gel electrophoresis and electrotransferred to 0.45 μm nitrocellulose membranes (Amersham; Buckinghamshire, UK) using a Bio-Rad semidry trans-blot, according to the manufacturer’s instructions. Membranes were blocked at 21 ± 1 °C for 1 h with PBS containing non-fat dry milk, 0.5% bovine serum albumin (BSA) and 0.2% Tween 20. Membranes were probed overnight at 4 °C using antibodies directed to SMN (1:5000) from BD Biosciences (Franklin Lakes, NJ, USA); Jagged1 (1:1000) and Delta1 (1:500) both from Santa Cruz Biotechnology (Santa Cruz, CA, USA); Notch and NICD (1:1000) both from Cell Signaling Technology (Danvers, MA, USA) and α-tubulin, (1:5000) from Sigma-Aldrich (St. Louis, MO, USA). Membranes were then washed with PBS and incubated for 2 h with the appropriate peroxidase-conjugated secondary antibody. Blots were developed with the chemiluminescent peroxidase substrate and visualized in chemiluminescence film (Amersham). The apparent molecular weight of proteins was determined by calibrating the blots with pre-stained molecular weight markers (Bio-Rad).

### 4.3. Mouse Model

Mouse lines were kindly provided by Dr. A. Burghes (The Ohio State University, Columbus, OH, USA). Experimental mice were obtained by breeding pairs of SMA carrier mice (*Smn^+/−^*; *SMN2^+/+^*; *SMNΔ7^+/+^*) on a FVB/N background. Identification of wild-type (WT) (*Smn^+/+^*; *SMN2; SMNΔ7*) and mutant SMA mice (*Smn^−/−^*; *SMN2; SMNΔ7*) was done by PCR genotyping of tail DNA as previously described [31,38]. WT and mutants mice were always used at P11. All experiments were performed according to the guidelines of the European Communities Council Directive for the Care of Laboratory Animals.

### 4.4. Immunofluorescence and Nissl Staining

Mice were anesthetized with 2% tribromoethanol (0.15 mL/10 g body weight, i.p.) and intracardiacally perfused with saline solution followed by 4% paraformaldehyde in 0.1 M phosphate buffer, pH 7.4. Spinal cords were post-fixed by immersion in the same fixative solution for 1–24 h. Then, transverse serial cryostat sections (10 μm thick) from lumbar segments were obtained with a Leica cryostat (Leica CM3050) and mounted on microscope slides. Sections were quenched with 3% H_2_O_2_ in phosphate buffer saline (PBS) and permeabilized with methanol for 5 min. Then, sections were blocked with 5% normal goat serum and 0.2% Triton X-100 in PBS for 1 h. Sections were incubated overnight at 4 °C with the primary antibody diluted in blocking solution. The following primary antibodies were used for immunofluorescence: anti-glial fibrillary acidic protein (GFAP) (1:1000) from Dako Cytomation (Glostrup, Denmark); anti-Jagged1 (1:200) and anti-Delta1 (1:200) from Santa Cruz Biotechnology; anti-Notch (1:200) from Cell Signaling Technology; anti-cleaved Notch1 (NICD) (1:200) from Santa Cruz Biotechnology; anti-neurofilament heavy chain (SMI-32, 1:1000) from Sternberger Monoclonals Inc. (Baltimore, MD, USA) and anti-neurogenin 3 (Ngn3) (1:200) from Millipore (Temecula, CA, USA). The SMI-32 antibody was used to specifically label spinal cord motoneurons as previously described [58].

For immunofluorescence, sections were incubated for 1 h with the appropriate secondary antibody, Alexa Fluor 555 goat anti-mouse IgG (1:200) or Alexa Fluor 488 goat anti-rabbit IgG (1:200) (Invitrogen, Carlsbad, CA, USA). Sections were then washed and mounted using Fluorescent Mounting Medium (Dako Cytomation). Immunohistochemical controls, performed by omitting the primary antibody, resulted in the abolition of the immunostaining. In some cases, spinal cord sections were also labeled with blue fluorescent Neuro Trace Nissl staining (Molecular Probes, Eugene, OR, USA).

### 4.5. Image Acquisition and Analysis

Images were acquired digitally using a 20× or 40× oil immersion objective with a Leica TCS SP2 confocal laser-scanning microscope. Images from WT and SMA mutant littermate preparations were taken with similar conditions (laser intensities and photomultiplier voltages). A minimum of ten lumbar spinal cord sections were studied for animal and experimental situation, with at least four mice for each experimental condition. In order to quantify GFAP immunoreactivity four selected fields of ventral spinal cord were digitized and the Mean Gray Value for GFAP immunoreactivity was measured in a blinded manner and corrected by the value of the area of the field to obtain the relative GFAP positive area. In order to quantify Jagged1, Delta1, Notch, NICD or Ngn3 immunoreactivities, four selected fields of ventral spinal cords containing cells labeled with anti-GFAP or SMI32 antibodies were digitized and the Mean Gray Value for Jagged1 or Delta1 immunoreactivity in GFAP-positive cells (astrocytes) as well as Notch, NICD or Ngn3 immunoreactivity in SMI32-positive cells (motoneurons) were measured in a blinded manner with ImageJ (W. Rasband, National Institutes of Health, Bethesda, MD; http://rsb.info.nih.gov/ij/). The values were background subtracted using the average Mean Gray Value of the preparation background in each of the experimental conditions and data were always represented as a percentage of the values in WT mice.

### 4.6. Statistical Analysis

All data are expressed as mean ± SEM values. Statistical significance was assessed by Student’s *t*-test. Differences were considered significant when the *p* value was less than 0.05.

## 5. Conclusions

In summary, our results demonstrate that, both *in vitro* and *in vivo*, SMN deficiency results in increased expression of Notch ligands on astrocytes and that lumbar spinal cord motoneurons of SMNΔ7 mice, adjacent to reactive astrocytes, display increased Notch signaling, as denoted by increased NICD levels and reduced expression of the proneural gene Ngn3. These findings may be relevant to understand some pathologic attributes of SMA motoneurons.
